# Baduanjin exercise for patients with knee osteoarthritis: A protocol for systematic review and meta-analysis

**DOI:** 10.1097/MD.0000000000022963

**Published:** 2020-10-30

**Authors:** Jieying Li, Shuai Yin, Ruiqing Li, Beibei Ge, Kaiqi Su, Xiaolei Song, Zhenhua Zhang, Yiniu Chang, Xiaodong Feng, Nan Wu

**Affiliations:** a Henan University of Chinese Medicine; b The First Affiliated Hospital of Henan University of Chinese Medicine, Zhengzhou, Henan, China.

**Keywords:** Baduanjin, knee osteoarthritis, meta-analysis, randomized controlled trial, systematic review

## Abstract

**Background:**

Knee osteoarthritis (KOA) is a common chronic degenerative disorder with an increasingly prevalence among the older individuals and the leading cause of pain in the elderly. Baduanjin, one of the ancient traditional Chinese mind-body exercise routine, has been recognized to have clinical benefits for KOA. We aim to evaluate the efficacy and safety of Baduanjin for patients with KOA through this systematic review and meta-analysis.

**Methods:**

Four English databases (Cochrane Central Register of Controlled Trials, PubMed, EMBASE, and Web of Science), and 4 Chinese databases (China National Knowledge Infrastructure, Chinese Biomedical Literature Database, Chinese Scientific Journal Database, and Wanfang Database), will be searched from establishment of the database until October 2020. The reference lists and the citation lists of studies meeting the inclusion criteria will also be searched to identify further studies for inclusion. The search languages are English and Chinese. The randomized controlled trials of Baduanjin training for patients with KOA will be included. The primary outcome will be assessed according to the Western Ontario and McMaster Universities Osteoarthritis Index. Meta-analysis will be conducted with the use of RevMan 5.3.

**Results:**

The results of this research will be submitted to a peer-reviewed publications.

**Conclusion:**

This systematic review aims to present evidence for whether Baduanjin training is an effective intervention which can improve both physical condition and life quality in patients suffering KOA.

**INPLASY registration number:**

INPLASY202090051.

## 1 Introduction

Knee osteoarthritis (KOA), one of the most prevalent chronic degenerative disorder involving the entire joint tissue,^[1]^ is the leading cause of pain and disability in the elderly.^[2]^ KOA is characterized by progressive loss of articular cartilage leading to pain, limited motor function, muscle weakness, joint stiffness, and swelling.^[3]^ Therefore, pain symptoms and physical function disability are the primary clinical symptoms of KOA.^[4–6]^ It is worth noting that the incidence of KOA among the elderly is relatively high, and approximately 30% to 50% of the elderly over 60 are affected by KOA.^[7,8]^ The overall pooled prevalence of symptomatic KOA in China was 14.6% in 2017,^[9]^ while the prevalence of symptomatic KOA presented a rapid growth trend.^[10]^ In addition, with disease progression, KOA not only might directly produces deleterious effects on individuals physical and mental health,^[11]^ but also reduces patients’ normal quality of life and increases the economic burden of patients’ families and social healthcare resources.^[12]^

Regarding the treatment management of KOA, the current treatment methods for KOA are mainly aimed at reducing joint pain and slowing down its progress. The main interventions to treat KOA including pharmacological therapy, non-pharmacological therapy, and surgery.^[13]^ As an effective therapy, surgery is not only expensive but also invasive. Although drug therapy can alleviate the symptoms of KOA, its long-term application is usually accompanied by certain side effects. Due to the cost issues and patient concerns regarding adverse effects causing by analgesic medications,^[14]^ clinical guidelines for the treatment of osteoarthritis of the knee emphasize nonpharmacologic treatments, mainly involving exercise, education, physical therapy, and weight loss, rather than the use of drugs or surgery.^[15]^ Traditional Chinese exercise, as one of the non-pharmacological therapy, has been used to treat KOA for a long time. It is gaining more and more attention owing to the characteristics of multi-target effects and fewer side effects.^[16]^ However, most traditional Chinese exercise, such as tai chi chuan and Wuqinxi, have some shortcomings, such as complicated movements, difficult learning, and long practice time, etc. The limitations of current KOA treatment necessitate further researches to discover the Cheaper, simpler, more efficacious, and safety treatments.

Baduanjin is a traditional Chinese mind-body exercise routine which combines rhythmic breathing, gentle movement, as well as meditation. As a common type of palliative low-impact and aerobic exercise, Baduanjin exercises could produce beneficial effects for KOA individuals by improving the pain, stiffness, balance ability, muscle strength, mobility, sleep quality, and cardiopulmonary function,^[17,18]^ and relieving psychological problems such as tension, depression, and anxiety.^[19]^ With the publication of several trials for KOA, evidences have certificated that Baduanjin has a good clinical effect.^[18]^ Although Baduanjin is a common method in managing KOA, there is still a lack of systematic review to summarize the efficiency of Baduanjin in treating KOA. Hereby, regardless of blinding, the purpose of the study is to systematically review current available randomized controlled trials (RCTs) that only use Baduanjin to treat patients with KOA to assess its efficacy and safety.

## 2 Methods

### 2.1 Registration

This systematic review protocol has been registered on the International Platform of Registered Systematic Review and Meta-analysis Protocols (INPLASY), and the registration number is INPLASY202090051 (https://inplasy.com/inplasy-2020-9-0051/). In this paper, the protocol will follows the recommendations of the methods introduced in the Cochrane Handbook 5.3 for Systematic Reviews of Intervention and reported according to the PRISMA-P guidelines.^[20]^ And if there are any changes, we will refine procedures described in this protocol and document the amendments in the INPLASY database and disclose them in future publications related to this meta-analysis.

### 2.2 Eligibility criteria

Eligibility criteria were detailed using the Participants, Interventions, Controls, Outcomes, and Studies framework.

#### 2.2.1 Types of participants

The subjects were patients with clinically confirmed KOA, being 45 years of age or older, regardless of any gender differences or ethnic background. However, patients will be excluded if they have other orthopedic problems of the knee, such as obvious knee joint deformity, joint locking unstable symptoms or any joint has been replaced, or if they are Combined with serious disease, such as stroke, heart disease, gout, or serious neurological disease and so on. Baseline is uniform for all participants in each RCT.

#### 2.2.2 Types of interventions

Studies were included if Baduanjin exercise was used as the sole intervention. Participants in experimental group should be treated with Baduanjin which has no restriction on types or training periods. Baduanjin training combined with other forms of therapy or other rehabilitation programs should be excluded.

#### 2.2.3 Types of controls

The control group should adopt one of the following treatment methods: A waiting list, health education, healthcare routine, self-help program, placebo, or no treatment. In addition, other similar behavioral exercise and physical therapy as controls should be excluded so as not to affect the accuracy of intervention results.

#### 2.2.4 Types of outcome measures

Main outcome(s): The score of the Western Ontario and McMaster Universities Osteoarthritis Index used to assess pain, stiffness, and physical functioning.

Additional outcome(s): the visual analog scale are used to measure pain degree. Some scales, such as knee injury and osteoarthritis outcome score, the isokinetic strength of the knee extensors, Berg Balance Scale, 6-minute walk test, active range of motion, and Lequesne & Mery Index, are used to test physical performance. While others, such as the Medical Outcomes Study Short Form-36, the Pittsburgh Sleep Quality of Index, and the 5 facet mindfulness questionnaire are used to assess quality of life.

#### 2.2.5 Types of studies

The eligible type of study is clinical RCT. And all relevant RCTs published in English and Chinese about Baduanjin for KOA will be included. Articles repeatedly published should be excluded.

### 2.3 Search methods for identifying the studies

#### 2.3.1 Information sources

We will perform medical retrieval in the following database: PubMed, EMBASE, Cochrane Library, Web of Science, National Knowledge Infrastructure, Wanfang Data Information Site, Chinese BioMedical Database, Chinese Science and Technique Journals Database.

#### 2.3.2 Searches strategy

The search terms are: (“Osteoarthritides” OR “Osteoarthrosis” OR “Osteoarthroses” OR “Arthritis, Degenerative” OR “Arthritides, Degenerative” OR “Degenerative Arthritides” OR “Degenerative Arthritis” OR “Arthrosis” OR “Arthroses” OR “Osteoarthrosis Deformans”) AND (“Baduanjin” OR “eight section brocades” OR “eight-section Brocade” OR “eight section brocades” OR “eight trigrams boxing” OR “eight-treasured exercises” OR “eight pieces of brocade” OR “Eight Brocade Section Baduanjin exercises” OR “Baduanjin exercise” OR “Baduanjin Qigong”). Chinese translations of these terms will be applied to Chinese database. Initially, and to increase the chance of identifying all relevant papers, the search will not be limited to any specific criteria. Articles published in English and Chinese will be considered. In addition, if necessary, depending on the specific situation, the reference list of the identified papers will also be searched. The proposed search strategy for PubMed is presented in Table 1.

**Table 1:**
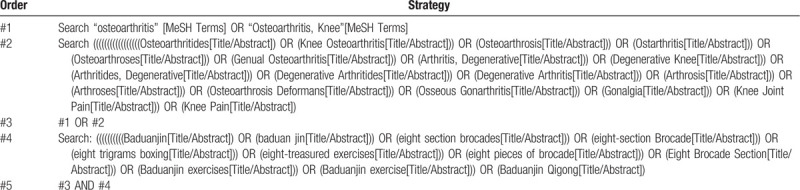
Search strategy (PubMed).

#### 2.3.3 Study selection

**Figure 1:**
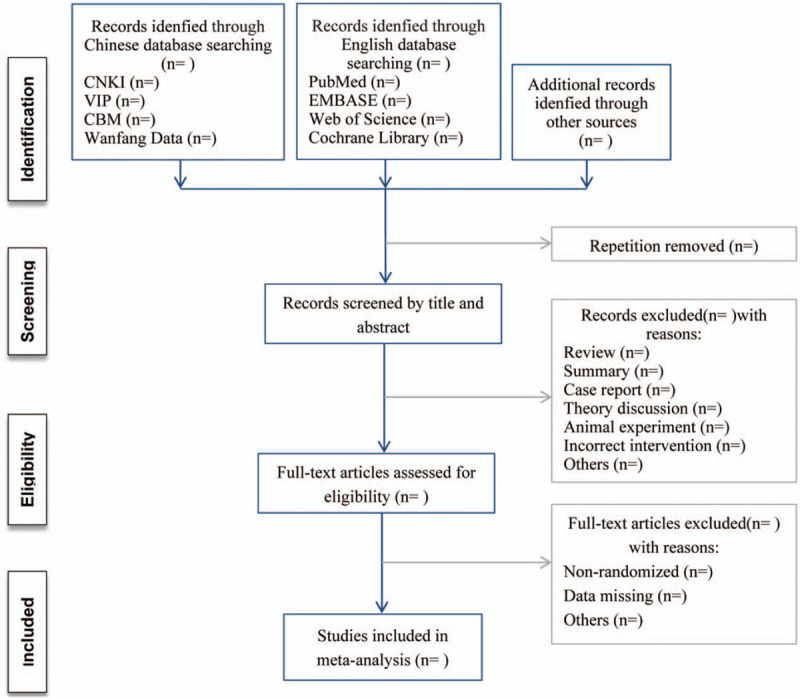
Flow diagram of study selection process.

The identified articles will be imported into the NoteExpress reference management software. After the initial removal of duplicate studies, 2 reviewers (Beibei Ge and Kaiqi Su) will independently screen titles and abstracts based on the eligibility criteria. For further assessment, full-text studies will be retrieved for all potentially meet the inclusion criteria. If studies contain insufficient information to make a decision about eligibility, to obtain further details, one reviewer (RuiQing Li) will try to contact authors of the original reports. During the procedure, reasons for exclusion should be noted and any disagreements should be solved by discussion until a consensus was reached. The specific process of study selection is shown in Figure 1.

#### 2.3.4 Data extraction

Two reviewers (Zhenhua Zhang and Yiniu Chang), will independently assess the eligibility of the studies retrieved against the inclusion and exclusion criteria. Then those studies meeting the criteria will be selected for use in the review. The following data will then be extracted from the studies selected for inclusion using a data collection form, and recorded in an Excel file. The following research characteristics will be extracted:

(1) Details of study: first author, year of publication, location of the study (country), study period or follow-up period, study design;
(2) Study population: age, gender, and sample size;
(3) Intervention characteristics: type, frequency, and duration;
(4) Outcome measures: Clinical effective rate, Western Ontario and McMaster Universities Osteoarthritis Index, visual analog scale, The knee injury and osteoarthritis outcome score, Study Short Form-36, 6-MWT, Berg balance scale, range of motion, Pittsburgh Sleep Quality of Index, the lower extremity functional scale, adverse effects, and so on.

### 2.4 Assessment of risk of bias

Two of our researchers (Shuai Yin and Xiaolei Song) will respectively use the bias risk tool (Cochrane Handbook for Systematic Reviews of Interventions 5.3) provided by the Cochrane Collaboration to evaluate the quality of the included literature. Seven domains should be evaluated, including random sequence generation, allocation concealment, blinding of participants and personnel, blinding of outcome assessment, incomplete outcome data, selective reporting of positive and/or negative findings, and other sources of bias. Among them, the “other sources of bias” included the following:

(1) whether the experimental design is practical;
(2) whether the baseline data is comparable;
(3) whether there is a clear conflict of interest leads to an increase in bias;
(4) whether there are clear inclusion and exclusion criteria.

Finally, we will make “Low risk,” “High risk,” and “unclear risk” judgments for each research literature, covering study limitations, inconsistencies, indirectness, imprecision, and publication biases. Then, a “risk of deviation” summary and a chart are generated to show the results. As with the previous process. If the 2 researchers differ in determining the bias, the differences are resolved through discussion. If there is still no consensus after discussion, we will seek advice from a third part (Xiaodong Feng and Nan Wu). Only literature with a score greater than 5 will be included.

### 2.5 Data analysis

#### 2.5.1 Date synthesis

Quantitative analysis was performed by meta-analysis using Cochrane collaboration software RevMan 5.3. The continuous variables are described using mean difference and 95% confidence interval between groups, whereas dichotomous data were presented as relative risk with 95% confidence interval.

#### 2.5.2 Assessment of heterogeneity

During the heterogeneity test, the Chi-squared test (the *x*^2^ test) was performed first, based on its finding, *I*^2^ statistic are applied to assess heterogeneity. The fixed-effect model is suitable to estimate the typical effect for studies when low heterogeneity (*I*^2^ < 50% and/or *P* > .10), whereas when substantial unexplained heterogeneity (*I*^2^ ≥ 50% and/or *P* ≤ .10), the random-effects model is applied to assess the average distribution for studies.

#### 2.5.3 Subgroup analysis

If significant levels of heterogeneity, or any incongruities, are detected within the analysis, subgroup analysis will also be employed to explore the possible causes of heterogeneity. Subgroup analysis will be based on possible factors that may lead to heterogeneity, such as intervention (different types of Baduanjin exercise), control (A waiting list, health education, self-help program, healthcare routine, placebo or no treatment, etc), ages (middle-age, old), the quality of study, treatment duration, and so on.

#### 2.5.4 Sensitivity analysis

We will conduct a sensitivity analysis to assess the robustness and reliability of the meta-analysis by eliminating low quality studies, if sufficient RCTs are available for our research. Besides, this will be achieved by assessing the impact of the sample size, high risk of bias, missing data, and selected models.

### 2.6 Confidence in cumulative evidence

The Grading of Recommendations Assessment, Development, and Evaluation system will be used for assessing the strength and the quality of the evidence. According to the grading system, the quality of evidence will be rated into 4 levels, as following: high, moderate, low, and very low.

## 3 Discussion

The pain, stiffness, and dysfunction of KOA directly reduce the quality of life. According to the recommendation of osteoarthritis research society international, many guidelines, KOA patients can take appropriate exercise intervention to relieve pain and improve functional ability.^[21,22]^ Traditional Chinese Medicine doctors have used Baduanjin exercise to accelerate the recovery of knee function for a long time, and have the advantages of simple operation, low price, easy to learn, and convenience. However, there is still a lack of corresponding multicenter randomized clinical controlled trials and more comprehensive meta-analysis. Since several recent clinical researches have focused on this promising treatment for KOA it is necessary to perform a high-quality systematic review and meta-analysis. Therefore, this review is expected to provide rigorous and objective evidences of the efficacy and safety of Baduanjin training for KOA.

## 4 Ethics and dissemination

The study will not contain any personal data and will not prejudice individual rights, so no ethical approval will be required. The study will be subject to rigorous peer review and may be published in a journal or circulated at relevant conferences.

## Author contributions

**Conceptualization:** Jieying Li, Shuai Yin, Ruiqing Li.

**Data curation:** Beibei Ge, Kaiqi Su, Zhenhua Zhang, Yiniu Chang.

**Formal analysis:** Shuai Yin, Xiaolei Song.

**Investigation:** Jieying Li, Shuai Yin, Kaiqi Su, Zhenhua Zhang.

**Methodology:** Shuai Yin, Xiaolei Song, Xiao-Dong Feng.

**Software:** Ruiqing Li, Kaiqi Su.

**Supervision:** Xiao-Dong Feng, Nan Wu.

**Validation:** Xiao-Dong Feng.

**Writing – original draft:** Jieying Li, Shuai Yin, Kaiqi Su.

**Writing – review & editing:** Jieying Li, Shuai Yin.
